# The relationship between health literacy and health‐seeking behavior amongst university students in Ghana: A cross‐sectional study

**DOI:** 10.1002/hsr2.2153

**Published:** 2024-05-23

**Authors:** Martin Gameli Akakpo, Maresa Neuerer

**Affiliations:** ^1^ Department of Psychology Regent University College of Science and Technology Accra Ghana; ^2^ Heidelberg Institute of Global Health University Hospital, Heidelberg University Heidelberg Germany

**Keywords:** health literacy, health promotion, health‐seeking behavior, prevention, self‐medication

## Abstract

**Background and Aims:**

The crises of the last decades have provided more evidence of the need for health literacy as a measure of resilience and preparedness. In this study the relationship between health literacy and health‐seeking behavior was investigated.

**Methods:**

This study used a cross‐sectional design with a questionnaire of five sections dedicated to health‐seeking behavior, health literacy, family background, socioeconomic status and demographics. Health‐seeking behavior was used in three dimensions namely preference for hospitals, self‐medication, and herbal medicine. The questionnaire was completed by 262 students at the University of Ghana.

**Results:**

A significant linear regression model (*R* = 0.39, *R*² = 0.15, Adjusted R² = 0.13, F = 8.89, *p* < 0.001) supported the relationship between health literacy and health‐seeking in health facilities such as hospitals. A Pearson correlation further showed an association between self‐medication and preference for herbal medication.

**Conclusion:**

Findings support the crucial role of health literacy in timely hospital visits by patients. This implies the need to improve health literacy through education, policy, and research. This can promote prevention of diseases through timely health‐seeking and improve preparedness against health crises. The study suggests health literacy should be integrated into educational curricula and regular health campaigns run by public health agencies.

## Key points

- Health literacy should be trained by health promoters.
- Health literacy aids patient resilience, prevention, and preparedness towards health crises.
- Health literacy should be part of school curriculum and regular public health campaigns.

## 1 INTRODUCTION

Previous research has pointed out the role of health literacy in the acceptance of health campaigns and adherence.^1^, ^2^ However, there is the need to pay more attention to the role health literacy plays in various types of health‐seeking behavior. Individuals can decide to self‐medicate, visit pharmacies, or go to the hospital. In many instances, this decision influences health outcomes.^3^, ^4^

In this paper we investigated how health literacy is related to the health‐seeking behavior of individuals. The health literacy considered in this paper is defined as knowledge about personal health situations and interpretation of health information to improve quality of life through efficient judgment.^5^, ^6^ Several studies such as Iskandar et al.^6^ and Miller^7^ have recommended more studies on health literacy and its role in health‐seeking and patient outcomes. Health literacy as a skill is important because it can help individuals prioritize their personal health and understand health information.^3^, ^8^ This information can be personal or public such as health warnings from the government and advice from health care professionals. Health literacy was therefore included in our study because of the support it has gained in both literature and clinical practice as a promoter of quality health decisions. According to our information, this is the first study looking at the relationship between health literacy and health‐seeking behavior among Ghanaian university students. As previous research shows health literacy and health‐seeking behavior strongly vary depending on the context, setting, and background of the studied population. Therefore, it will be interesting to investigate this relationship among university students in the capital city of Ghana.

Health‐seeking behavior has been recommended in many studies as a contributor to health outcomes and represents a complex behavioral phenomenon.^9^, ^10^ It has also been recommended by many health experts and researchers as safer than ignoring symptoms or avoiding regular health checks. Health‐seeking behavior includes action taken by individuals, who are under the impression to have a health condition, “to prevent, minimize or cure a disease condition as well as maintain a good health status” [11, pp. 841]. Thereby, it represents a response to perceived discomfort or symptoms.^11^

The two variables included in this study, health literacy and health‐seeking behavior, are important in modern patient centered healthcare.^7^, ^9^, ^12^ This is because of the numerous opportunities and risks which have either existed or are forming in the last few years. These stem from existing health risks, increased mobility, abundance of information through the internet and lifestyle changes associated with increased technology use, and globalization amongst others. It is important for researchers to keep up to date with trends in human activity to ensure important variables are investigated with varied samples. The findings of this study will empower healthcare workers, patients, and researchers with insights needed to improve health literacy and understand how it influences willingness to access the different avenues for care. Findings will also benefit policy makers and public health educators through information on the public knowledge among university students which could serve as indicators for future health campaigns or policy.

### 1.1 Health literacy

Health literacy has been identified as a facilitator of public health targets^13^ and personal health outcomes.^6^, ^14^ The ability of individuals to understand and comply with health prevention campaigns can be aided by this skill.^1^, ^12^ Adherence to these campaigns can save health systems significant budget, time, and effort needed to deal with epidemics.

Beyond prevention, health literacy can regulate treatment and medication adherence after visits to healthcare providers.^2^, ^13^ Adherence is important for many health conditions including medication dosage and timing. Some conditions can be managed with lifestyle changes which may be difficult for individuals. However, an understanding of the consequences of non‐adherence can lead to an uptake of the changes recommended by the physician or other care providers.^3^ An instance can be lifestyle changes required for young individuals at high risk of developing hypertension. If the consumption of alcohol, high cholesterol diets, high sodium diets, and a sedentary lifestyle are all entrenched in their lives, making changes may be difficult. Understanding the risks of severe hypertension, the elevated risk of stroke and heart attack can help such a person adhere to their physician's recommendation. Such adherence increases the likelihood of managing or even preventing many health conditions and this in turn reduces the burden on the health system.^5^ In addition to aiding prevention and adherence, health literacy in the population can aid in crisis response to epidemics and other public health emergencies.^1^

As witnessed during the covid‐19 pandemic, the success of these response measures is partly dependent on adherence from the public. The adherence can be influenced by their understanding of transmission risks to themselves, the family, and members of their community.^8^ With health literacy individuals can understand that they could be agents of distribution and that can lead to unpleasant consequences for themselves and people they care about. This act of taking responsibility can break a chain of infections and save the individual a lot of time and resources while reducing burden on the health system. This in turn helps public health officials to meet control targets.

From the three scenarios provided above, that is, prevention, treatment adherence, and crisis control, health literacy is a useful skill which should be promoted in every health system. There is evidence of condition related health literacy campaigns which have helped many health systems including Ghana's. Relatively successful campaigns like those run for tuberculosis and HIV/AIDS are good examples. A high level of health literacy can aid resilience and preparedness for crises.^1^, ^6^ This may reduce the amount of time needed for such large‐scale emergency measures. A low level of health literacy means new resources need to be mobilized to educate the public on every condition of concern.^3^, ^13^ This increases the response time, infection risks and budgeting requirements.

In a study by Kugbey et al.^2^ in Ghana 205 women living with breast cancer were interviewed. The study measured the influence of health literacy and health information on the health‐related quality of life of these patients. Health‐related quality of life included satisfaction, anxiety, and depression. Kugbey et al.^2^ found that health literacy improved the quality of life of these patients. Health literacy helped patients with access to information to manage their anxiety and depression levels while adhering to their treatment regimens.

Health literacy and health‐seeking behavior are closely related.^13^ This means individuals with a higher health literacy know when and where to seek care. This relationship is important throughout prevention, adherence, and crisis management. Timely visits to health care centers can avert dangerous escalation of health conditions.^6^ This literacy can also improve trust in hospitals and influence people to seek care with well trained professionals such as doctors and nurses. Health systems benefit from timely health‐seeking decisions, adherence to prevention and treatment protocols, and an understanding of individual roles in crisis control. All these ‘benefits’ can be influenced by health literacy.^1^, ^12^

### 1.2 Health‐seeking behavior

Health‐seeking behavior is directly linked to the utilization of healthcare services and influenced by a diversity of factors, such as the physical and social environment including societal norms and values, culture, religion, socioeconomic status, psychological and demographic factors, as well as health system characteristics and access to services.^15^, ^16^, ^17^ Based on the individually perceived severity of a condition or disease, a patient might decide to seek care.^10^ Additional important factors for healthcare‐seeking behavior encompass the timing and type of healthcare provider approached, which influence the eventual health outcome.^18^ Furthermore, health‐seeking behavior gives insights into factors enabling or preventing people from making choices benefiting their health status as well as insights into health‐related values and attitudes towards healthcare services.^10^, ^19^ This information is critical for the planning of healthcare systems and reaching patients with the right care at the right place and time.

In many societies including Ghana, the avenues for seeking care are numerous and range from different levels of allopathic to traditional healthcare providers. Individuals can consult university trained professionals, home‐trained attendants, or apprenticeship trained traditional workers. These groups use different approaches with the same aim to create a favorable health outcome for their patients. Medication can be bought on prescription or over‐the‐counter preceded by a visit to a healthcare facility or based on self‐diagnosis.

Several studies have investigated health‐seeking behavior and its predictors in sub‐Saharan African countries, such as among children in Malawi, rural dwellers in under‐resourced communities in Ghana, and Ghanaian women to explore the association between their healthcare decision making capacity and health‐seeking behavior for their children.^15^, ^16^, ^20^ Appropriate health‐seeking behavior of parents for their sick children can be critical to reduce child morbidity as poor healthcare utilization can lead to an increased morbidity and mortality rate among children even though many programs have been developed to improve child health.^21^ Moreover, the appropriate health‐seeking behavior leads to the timely and effective treatment of diseases and thus contributes to a reduction in disease severity.^22^ A study by Jin et al.^23^ stresses the importance of health workforce availability on the accessibility of healthcare services, particularly outpatient visits, and shows a relationship between health workforce availability and health‐seeking behavior.

Other studies have particularly looked at health‐seeking behavior among university or college students in Nigeria, the US, or India for example.^24^, ^25^, ^26^ However, in Ghana students’ health‐seeking behavior was only investigated in a resource‐poor setting.^27^ The relevance of this study lies in the lack of research on health literacy and health‐seeking behavior among Ghanaian university students in the city of Accra and has the potential to give insights into the utilization of healthcare services in this specific setting.

### 1.3 Research questions

The aim of this study is to find out whether a relationship exists between health literacy and different forms of health‐seeking behavior, namely health‐seeking in hospitals, a preference for self‐medication with over‐the‐counter drugs, and the use of herbal medication.

## 2 MATERIALS AND METHODS

### 2.1 Study design

A cross‐sectional survey was employed for this study. The questionnaire used standard scales to measure health literacy, and health‐seeking behavior. Age, sex, and health insurance status were also measured. Questionnaires were distributed to students after researchers explained the purpose of the research and the rights of participants. All participants were then given 15 min to complete the questionnaires and return it to the researchers.

### 2.2 Setting

The study was conducted at the University of Ghana, in the Ghanaian capital of Accra. Accra is the most affluent city in Ghana with high exposure to health information, specialist hospitals, pharmacies, and herbal medicine services. Access to all forms of health‐seeking considered in this paper, that is health facilities, self‐medication through pharmacies and seeking herbal medications is relatively higher in Accra than in other cities in Ghana. Students in the city of Accra thus have easy access to all health facilities and products as well as health information from regular awareness campaigns.

### 2.3 Participants

The criteria for inclusion in the study included enrollment at the University of Ghana and being at least 18 years old. All participants were fluent in the English language and were enrolled in degree programs taught in English. All individuals were asked whether they wanted to participate in the study. After their confirmation they were informed about their right to ask questions and opt out whenever they desired.

### 2.4 Variables

The study used health and health‐seeking behavior as the main variables of interest. Health‐seeking behavior was split into three components. These were seeking health at health facilities, including hospitals and clinics, self‐medication by using medication at home or over the counter medication from pharmacy, and seeking health by using herbal medication. Health insurance status, socioeconomic status, level of education, age, and sex were measured.

### 2.5 Data sources/measurement

Data collection was done with a questionnaire split into five sections namely health literacy, health‐seeking behavior, family background, socioeconomic status, and demographics. Health literacy was measured with a 16‐item scale European Health Literacy Survey Questionnaire (HLS‐EU‐Q16), developed by a European consortium of literacy.^28^ This scale has been used in many settings throughout the world.^29^ Health‐seeking behavior was measured with a scale developed by Nuhu.^30^ The sub‐scales presented eight items each to measure the three dimensions of health‐seeking considered in this work, namely visiting health facilities, use of self‐medications and use of herbal medicine. The section for family background asked about the health insurance status of the participant's family. The fourth section focused on socioeconomic status using questions about the participant's personal health insurance status, amount of money they spent in a day, and the level of education of their parents. The final section which was demographics asked about the sex and age of participants. Data from the questionnaires were inputted in SPSS version 26^31^ and analyzed.

### 2.6 Bias

Participants who were enrolled in medicine, nursing, and other health science fields were excluded from the study. This was to avoid bias with regard to their health literacy and easier access to hospitals where they are students.

### 2.7 Sample size

Using a convenience sampling of university students, 262 students filled in the questionnaires. Using G ‐ Power 3.1* with an a priori power analysis with a linear regression as the selected statistical test, a minimum sample size estimate of 74 was recommended. This was based on an effect size (f²) of 0.15 and a power (1 ‐ **β** error probability) of 0.95, supported by Beck.^32^ Researchers recruited beyond the minimum to the strength of results and based on recommendations of Kang^33^ and Hagan and colleagues.^34^ The sample size of 262 was therefore deemed sufficient.

### 2.8 Statistical analysis

Both univariate and multivariable statistical analysis were conducted. To comply with best reporting standards, recommendations of Assel^35^ were followed. Statistical analysis was done with SPSS version 26. To allow consistency, only complete responses were included in final computations. Scale scores were included in final computations if all items on that scale were complete. Univariate analysis was computed to understand the demographic characteristics of participants and the range of scores on key variables. These were means, standard deviations, and minimum to maximum scores (see Table 1). Multivariable analysis was conducted using Pearson correlation coefficients (2‐tailed) and a multiple linear regression model. As part of the regression, model diagnostics were computed with the cooks distance, durbin watson test, tolerance and VIF scores. The correlations, Chronbach alpha reliability coefficients (see Table 2), and the regression (see Table 3) used health literacy and all three dimensions of health‐seeking behavior.

**Table 1:** Descriptive table of responses to family background, and socioeconomic status.

	Frequency	Percentage
***Family Health Insurance status***	*N* = *256*	*100%*
No Health Insurance	18	7.03
Private Health Insurance	30	11.72
Government Health Insurance (NHIS)	208	81.25
***Personal Health Insurance status***	*N* = *256*	*100%*
No Health Insurance	32	12.5
Private Health Insurance	22	8.6
Government Health Insurance (NHIS)	202	78.9
***How much money do you spend in a day?***	*N* = *240*	*100*
Low (Less than GHC 12 per day	50	20.83
Average (Between GHC 24 and GHC 120 per day)	172	71.67
High (Greater than GHC 120 per day)	18	7.5
**Highest level of most educated parent**	*N* = *256*	*100%*
No Formal Education	12	4.7
Basic Education	21	8.2
Senior High School	78	30.5
Bachelor's Degree	70	27.3
Masters	53	20.7
Ph.D. (Doctorate)	22	8.6

*Note*: Demographic features of respondents. N, Sample size.

**Table 2:** Summary of score ranges, means, standard deviations, reliabilities and correlations of health literacy and dimensions of health‐seeking behavior.

Variables	L/H	M	SD	1.	2.	3.	4.
1. Health Literacy	16/64	47.10	8.08	(0.88)			
2. HSB ‐ Facilities	10/40	28.68	5.89	0.29^a^	(0.85)		
3. HSB ‐ Self Medication	8/54	21.41	6.16	0.06	−0.12	(0.72)	
4. HSB ‐ Herbal Medicine	8/40	15.42	6.81	−0.04	−0.06	0.27^a^	(0.94)

Abbreviations: HSB = Health‐Seeking Behavior, L/H = Lowest Score/Highest Score, M = Mean, SD = Standard Deviation. () = Cronbach alpha reliability coefficient.

^a^ = Significant.

**Table 3:** Coefficients of model using health literacy as the outcome with health‐seeking behavior as predictors.

Variables	Unstandardized *B*	Standard Error	*Standardized Coefficients β*	Sig (p)	95% CI Lower Bound	95% CI Upper Bound
Constant	27.76	4.09		.00	19.69	35.84
HSB‐ Facilities	.56	.11	.38	.00	.34	.77
HSB ‐ Self Medication	.12	.10	.09	.23	‐.08	.33
HSB ‐ Herbal Medicine	.01	.09	.01	.94	‐.17	.19

*Note*: ***B*** = Unstandardized B, ***β*** = Standardized Coefficient Beta, Sig (p) = Significance, HSB = Health‐Seeking Behavior, 95% CI = 95% Confidence Interval.

### 2.9 Ethical considerations

Principles of informed consent, confidentiality and anonymity were adhered to in this project. All participants were educated on their rights and the purpose of the research before questionnaires were handed out to those who agreed to participate. All participants were assured that data will be handled confidentially and only by the research team. To guarantee anonymity, researchers did not collect personal identification data such as names, email addresses, phone numbers and student identification numbers. Data collection was anonymous, and no experimental manipulations were involved.

## 3 RESULTS

### 3.1 Participants

There were 262 participants in this study. They were aged between 18 and 34 with a mean age of 21.91 and a standard deviation of 2.21. There were 204 females (77.9%), 53 (20.2%) males and 5 participants did not complete the sex question.

### 3.2 Main results

Multivariable statistics were used to investigate the relationship between all the variables included in the regression model. To show the reliability of the scales, Cronbach alpha reliability coefficients were computed as presented in Table 2.

To test the relationship between health literacy and all three dimensions of health‐seeking behavior, a model with health literacy as the outcome and health‐seeking behavior as predictor was used. As part the regression, tests for effects of each observation, autocorrelation, and collinearity were conducted. A cooks distance range between 0.00 and 0.10, showed no cause for concern. A durbin watson test score of 1.63 indicated there was no alarming autocorrelation. Collinearity statistics indicated no alarming multicollinearity as indicated by tolerance values between 0.92 and 0.99 and variance inflation factors (VIF) between 1.01 and 1.09. These figures are acceptable as suggested by Weisburd and Britt.^36^

A significant model emerged (*R* = *.39, R²* = *.15, Adjusted R²* = *.13, Standard Error* = *7.79, effect size f* ^*2*^ = *0.18*). An ANOVA computed as part of the model showed that differences in scores on health literacy were related to scores on health‐seeking behavior (*F* = *8.89, degrees of freedom* = *3, 152, p* < *0.001*). A table of coefficients showed that visiting hospitals, clinics and health centers was the only dimension which had a significant relationship with health literacy. Confidence intervals for the predictors are provided in the same coefficients table (*see* Table 3).

### 3.3 Summary of results

In summary, the results show a relationship between scores on health literacy and health‐seeking in health facilities, namely hospitals, clinics, and health centers. A relationship between self‐medication or use of herbal drugs and health literacy was not supported. However, there was a positive correlation between self‐medication and use of herbal drugs.

## 4 DISCUSSION

The findings from this work can be summarized in two main points. These are the relationship between health literacy and visiting health facilities, and the relationship between self‐medication and herbal medicine preference. It is important to interpret the absence of a relationship between health literacy and other forms of health‐seeking behavior used in this study.

Firstly, the suggested relationship between health literacy and preference for health facilities as a dimension of health‐seeking behavior is equivalent to the findings of Amoah et al.^13^ This relationship means individuals who are more knowledgeable and decisive about their health are more likely to seek help in hospitals, clinics, or health centers. These individuals recognize the importance of structured or test‐driven investigation and treatment of health issues. In Ghana and many other health systems, hospitals, clinics, and health centers have tried and tested protocols for the treatment of health issues. The decentralization of decision making ensures and division of labor amongst departments in these health facilities improves the confidence of individuals who are health literate. Health literates can recognize when they need to visit the hospital and why they must adhere to their medication regimes. This competence allows them to rely on hospitals for their health needs. Based on these findings it can be deduced that individuals with high health literacy trust health facilities more than other avenues of healthcare. This could be because the processes and procedures in these facilities are well established, transparent, and verifiable. After visiting a healthcare provider, individuals with a high level of health literacy can read online about the medication they were given or the tests they were asked to run. Asking for second opinions is usually not difficult because practitioners in other facilities can speak to these tests and procedures based on known protocols and their training. Health literates can thus be confident about the care they receive because the outcome is verifiable, and their skills will allow them to interact with healthcare providers about the care they are receiving.

The lack of support for a relationship between health‐seeking behavior through self‐medication and the preference for herbal medicine is not surprising, as reported by Cobbold and Morgan.^37^ This is because health literacy shifts preference to a style of healthcare which is underpinned by tests, specializations, and principles of understanding the exact cause of a condition. This finding can further be explained by the relatively easy access students have to hospitals on and close to their campus. Additionally, from the results (see Table 1) most of the students were on health insurance and this grants them relatively easy access to hospital care. The attempt to know the exact cause then motivates a treatment of that exact root cause. Self‐medication and herbal medicine in the Ghanaian context are rather management techniques used for conditions which may not be deemed serious.^2^

Secondly, the correlation between self‐medication and the preference for herbal medicine as dimensions of health‐seeking behavior is understandable. This corresponds to the findings of Cobbold and Morgan.^37^ The relationship means individuals who prefer to buy over‐the‐counter medications from pharmacies and use them without visiting hospitals may also opt to visit herbal medicine providers when needed. In Ghana herbal medicine is easily accessible with vendors publicizing the potency of their products for the most common conditions on university campuses and many streets in the city. In the current digital age, many herbal medicines service providers upload videos of their products online and actively advertise them on television and radio. Individuals who do not want to visit a hospital will rely on such content to acquire the medications from the pharmacy or buy the herbal concoctions from a nearby counter for use. These individuals most likely lack an understanding of the possible side‐effects of these unregulated herbal medications. Additionally, an insufficient understanding of the need to use medications after the exact cause of an illness is known through tests may drive the unregulated use of over‐the‐counter medications bought at pharmacies or herbal medications.

In summary, the findings suggest health literacy as a useful competence which helps individuals make health decisions in line with health campaigns and goals of health systems. Health campaigns in Ghana and many other health systems have encouraged timely hospital visits, adherence to safety protocols and avoidance of self‐medication. Findings from this study show that health literacy is a competence which can aid the uptake of health campaigns. The skill encourages the patronage of hospitals and the use of medications for known conditions. The implications of these findings are discussed in the next section.

### 4.1 Practical implications

Findings from this work have implications for health educators, policymakers, and researchers.

For educators, the suggestion of a relationship between health literacy and health‐seeking in hospitals means a focus on health literacy is important. Instead of focusing on disconnected campaigns aimed at specific health conditions, public health educators can drive sustained health literacy through many avenues. These include social gatherings, schools, and other opportunities which have hitherto been used to educate the public on specific topics. A more sustainable approach will be an established routine for training health literacy through school and other informal gatherings. Topics such as blood pressure, nutrition, effects of medication, types of hospital tests, and exercise could be treated without waiting for health emergencies like tuberculosis, HIV/AIDS, and covid‐19. These emergencies typically lead to painful drives to convince the public to visit the hospital, and this can result in some resistance and other non‐adherent behaviors common with such emergencies. This point leads to the second implication which concerns policymakers. Educators should view health literacy as a basis for the success of health campaigns and a way to empower the public towards a more well‐informed adherence and prevention‐oriented behavior.

Policy makers should include health literacy in school curriculum, civic education, and law. In school, students usually take physical education and sports lessons without receiving deeper explanations of the benefits of exercise, or concepts such as blood pressure. This can be addressed by training teachers to provide health‐oriented lessons which should be examined in future research. Policy makers can also include health literacy in regular civic education drives and literature which speaks about the responsibilities and rights of individuals. This approach will create a more resilient and prepared public who will know how to prevent or even respond to health emergencies without the need for coercive measures like was witnessed during the covid‐19 pandemic. Such readiness increases the likelihood that avoidable emergencies will be prevented while the losses from unavoidable emergencies will be kept to a minimum.

Finally, it is recommended that researchers investigate how to improve health literacy in the public with the intention of making health campaigns easier and improving general wellbeing. Research projects should develop clear learning objectives and programs which can be included in curricula and health promotion campaigns regularly run by public health professionals. Researchers in education, health, and civic affairs should coordinate to drive health literacy. It will promote a healthier society which is more resilient to the regular wave of pandemics, epidemics, and other health crises. Researchers have the skill to develop content and campaigns with long‐term objectives driven to improve public knowledge and drive policy. Such work will be done without the pressure of health crises which usually drive fast paced mitigation efforts. A health literate population will understand the need to prevent illness through time‐tested methods learnt in school or other gatherings. They can respond faster and better to government calls for extraordinary measures aimed at containing novice diseases.

In summary, a health literacy drive by health educators, policymakers and researchers can result in a more health‐conscious population at every level from school children to health workers. Such a focus on health awareness will create a resilient and prepared society which would not need new sophisticated campaigns for healthcare topics. Rather the society will only need reminders and updated information which will require less resource deployment and reduce the risk of non‐adherence.

### 4.2 Limitations

This study has two limitations, specifically the use of a sample from one university and use of only adults in university settings. Health literacy is relevant to every demographic group in the population. Future studies can use a broader sample to improve the generalizability of the findings. The use of a sample drawn from more universities and people not enrolled in any university will make the findings more generalizable. Using the sample of this study, findings are only generalizable to university students or adults with similar demographic properties such as education, age and level of health literacy in a capital city like Accra.

## 5 CONCLUSION

This study contributes to literature about the role health literacy can play in health‐seeking behavior. The findings provide evidence which can drive education campaigns and policy to improve health literacy amongst the public. Researchers can take cues from the work and replicate this design with other samples as well as use the recommendations from the work to conduct more studies. Using data from a young population in Ghana, the study provides recommendations which can shape new health literacy drives in Ghana and other countries with similar demographics. As the world tries to recover from a difficult covid‐19 pandemic, studies like this serve as a reminder of the need to build resilient societies. This resilience is not solely about budgetary readiness or financial reserves but more about a society which is aware of a wide range of health conditions and knows when or where to seek help. As demonstrated by the current study, such readiness can be built through coordinated focus by health educators, policymakers, and researchers.

## AUTHOR CONTRIBUTIONS

**Martin Gameli Akakpo**: Conceptualization; Investigation; Methodology; Writing—original draft; Writing—review & editing; Formal analysis; Project administration; Supervision; Data curation. **Maresa Neuerer**: Conceptualization; Writing—review & editing; Writing—original draft; Methodology; Formal analysis. Both authors have read and approved the final version of the manuscript. Martin Gameli Akakpo had full access to all of the data in this study and takes complete responsibility for the integrity of the data and the accuracy of the data analysis.

## CONFLICT OF INTEREST STATEMENT

The authors declare no conflicts of interest.

## ETHICS APPROVAL

IRB approval was not required for this study because of the anonymous nature of data collection. No harmful variable was introduced in our cross‐sectional survey design. However, academic superiors of all authors were informed about the conduct of this study. All participants in this research read an informed consent statement. All participants were informed of their right to refuse participation and withdraw from the study at any time.

## TRANSPARENCY STATEMENT

The lead author Martin Gameli Akakpo affirms that this manuscript is an honest, accurate, and transparent account of the study being reported; that no important aspects of the study have been omitted; and that any discrepancies from the study as planned (and, if relevant, registered) have been explained.

## Data Availability

The data that support the findings of this study are available from the corresponding author upon reasonable request. Additional data not presented in the results or appendix of this article is available from the corresponding author upon reasonable request. Data from this study are available upon reasonable request to the corresponding author.
